# Complete Genome Sequence of a Giant Sea Perch Iridovirus in Kaohsiung, Taiwan

**DOI:** 10.1128/genomeA.01759-15

**Published:** 2016-04-28

**Authors:** Chiu-Ming Wen, Jiann-Ruey Hong

**Affiliations:** a Department of Life Sciences, National University of Kaohsiung, Kaohsiung, Taiwan; b Institute of Biotechnology, National Cheng-Kung University, Tainan, Taiwan

## Abstract

We report here the complete genome sequence of a megalocytivirus strain, GSIV-K1, isolated from a farmed giant sea perch (*Lates calcarifer*) in Kaohsiung, Taiwan. GSIV-K1 causes mortality in farmed marine fish, including giant sea perch and groupers. The genome sequence is nearly identical to the genome of the orange-spotted grouper iridovirus.

## GENOME ANNOUNCEMENT

*Megalocytivirus*, a genus of fish viruses within the family *Iridoviridae*, causes emerging infectious iridovirus diseases that can result in moderate-to-heavy losses in various species of both cultured and wild freshwater and marine fish (1). *Megalocytivirus* contains 3 major subgroups, the infectious spleen and kidney necrosis virus (ISKNV), Red Sea bream iridovirus (RSIV), and turbot reddish body iridovirus (TRBIV) (2). RSIV is included on the list of notifiable terrestrial and aquatic animal diseases compiled by the World Organisation for Animal Health (http://www.oie.int/en/animal-health-in-the-world/oie-listed-diseases-2015/).

In June 2006, a virus designated GSIV-K1 was isolated from a diseased giant sea perch (*Lates calcarifer*) farmed in Kaohsiung, Taiwan (3). The nucleotide sequences of the major capsid protein suggest that GSIV-K1 belongs to the RSIV group (2, 3). GSIV-K1 has a sequence that is identical to that of the orange-spotted grouper iridovirus (OSGIV) genes of ORF35L and ORF39L (4). To obtain the complete genome sequence of GSIV-K1, the virus was cultured on SPB cells (5), and the DNA from purified viral particles was extracted using a WelPrep DNA kit (Welgene Biotech), according to the manufacturer’s instructions. For library construction, 10 µg of total DNA was sonicated using a Misonix 3000 sonicator for sizes ranging from 400 to 500 bp. DNA sizing was performed with a bioanalyzer DNA 1000 chip (Agilent Technologies). Sonicated DNA (1 µg) was end repaired, A-tailed, and adaptor ligated, according to the Illumina TruSeq DNA preparation protocol. ConDeTri was implemented for trimming and removal of the reads according to the quality score (6). The total number of high-quality paired-end sequence reads after trimming were 17.66 million. Cleaned and filtered nuclear reads were assembled *de novo* using ABySS (7). Gene predictions were performed using GeneMarkS (8) and annotation based on BlastX results. The resulting predictions were searched against the NCBI nonredundant database using Blastp (http://blast.ncbi.nlm.nih.gov/Blast.cgi).

The circular genome of GSIV-K1 comprises 112,565 bp and has 135 putative open reading frames. A phylogenetic analysis of the genome sequence demonstrated that GSIV-K1 is nearly identical to OSGIV, followed by rock bream iridovirus, RSIV, large yellow croaker iridovirus, ISKNV, and TRBIV.

### Nucleotide sequence accession number.

The complete genome sequence of GSIV-K1 has been deposited in GenBank under the accession no. KT804738.

## References

[1] Kurita J, Nakajima K 2012 Megalocytiviruses. Viruses 4:521–538. doi:10.3390/v4040521. 22590684 PMC3347321

[2] Fu X, Li N, Liu L, Lin Q, Wang F, Lai Y, Jiang H, Pan H, Shi C, Wu S 2011 Genotype and host range analysis of infectious spleen and kidney necrosis virus (ISKNV). Virus Genes 42:97–109. doi:10.1007/s11262-010-0552-x. 21107672

[3] Wen CM, Lee CW, Wang CS, Cheng YH, Huang HY 2008 Development of two cell lines from *Epinephelus coioides* brain tissue for characterization of betanodavirus and megalocytivirus infectivity and propagation. Aquaculture 278:14–21. doi:10.1016/j.aquaculture.2008.03.020.

[4] Wen CM, Ku CC, Wang CS 2013 Viral susceptibility, transfection and growth of SPB—a fish neural progenitor cell line from the brain of snubnose pompano, *Trachinotus blochii* (Lacépède). J Fish Dis 36:657–667. doi:10.1111/jfd.12067. 23305502

[5] Wen CM, Wang CS, Chin TC, Cheng ST, Nan FH 2010 Immunochemical and molecular characterization of a novel cell line derived from the brain of *Trachinotus blochii* (Teleostei, Perciformes): a fish cell line with oligodendrocyte progenitor cell and tanycyte characteristics. Comp Biochem Physiol A Mol Integr Physiol 156:224–231. doi:10.1016/j.cbpa.2010.02.003. 20167281

[6] Smeds L, Künstner A 2011 ConDeTri—a content dependent read trimmer for Illumina data. PLoS One 6:e26314. doi:10.1371/journal.pone.0026314. 22039460 PMC3198461

[7] Simpson JT, Wong K, Jackman SD, Schein JE, Jones SJM, Birol İ 2009 ABySS: a parallel assembler for short read sequence data. Genome Res 19:1117–1123. doi:10.1101/gr.089532.108. 19251739 PMC2694472

[8] Besemer J, Lomsadze A, Borodovsky M 2001 GeneMarkS: a self-training method for prediction of gene starts in microbial genomes. Implications for finding sequence motifs in regulatory regions. Nucleic Acids Res 29:2607–2618. doi:10.1093/nar/29.12.2607. 11410670 PMC55746

